# Assessment of regression-based methods to adjust for publication bias through a comprehensive simulation study

**DOI:** 10.1186/1471-2288-9-2

**Published:** 2009-01-12

**Authors:** Santiago G Moreno, Alex J Sutton, AE Ades, Tom D Stanley, Keith R Abrams, Jaime L Peters, Nicola J Cooper

**Affiliations:** 1 Dept of Health Sciences, University of Leicester, Leicester, UK; 2 Dept of Community Based Medicine, University of Bristol, Bristol, UK; 3 Dept of Economics, Hendrix College, Conway, Arkansas, USA

## Abstract

**Background:**

In meta-analysis, the presence of funnel plot asymmetry is attributed to publication or other small-study effects, which causes larger effects to be observed in the smaller studies. This issue potentially mean inappropriate conclusions are drawn from a meta-analysis. If meta-analysis is to be used to inform decision-making, a reliable way to adjust pooled estimates for potential funnel plot asymmetry is required.

**Methods:**

A comprehensive simulation study is presented to assess the performance of different adjustment methods including the novel application of several regression-based methods (which are commonly applied to detect publication bias rather than adjust for it) and the popular Trim & Fill algorithm. Meta-analyses with binary outcomes, analysed on the log odds ratio scale, were simulated by considering scenarios with and without i) publication bias and; ii) heterogeneity. Publication bias was induced through two underlying mechanisms assuming the probability of publication depends on i) the study effect size; or ii) the p-value.

**Results:**

The performance of all methods tended to worsen as unexplained heterogeneity increased and the number of studies in the meta-analysis decreased. Applying the methods conditional on an initial test for the presence of funnel plot asymmetry generally provided poorer performance than the unconditional use of the adjustment method. Several of the regression based methods consistently outperformed the Trim & Fill estimators.

**Conclusion:**

Regression-based adjustments for publication bias and other small study effects are easy to conduct and outperformed more established methods over a wide range of simulation scenarios.

## Background

Publication bias (PB) has the potential to distort the scientific literature [1,2]; since it is the "interest level", or statistical significance of findings, not study rigour or quality, that determines which research gets published and is subsequently available [3]. A meta-analysis of the published literature will be biased and may adversely affect decision making if PB exists.

More generally, the tendency for smaller studies to show a greater effect than larger studies, when evaluating interventions, has been named small-study effects [4]. These could be due to publication bias, or other factors. Any factor which confounds the relationship between-study effect and study size may cause small-study effects. For example, if an intervention is more effective in high-risk patients, and the small studies are, on average, conducted in higher-risk patients, this may result in a larger treatment efficacy being observed in the smaller studies. Further, it has been observed that certain aspects of trial quality influence effect size estimates and empirical evidence suggests that small studies are, on average, of lower quality [4].

**Figure 1:**
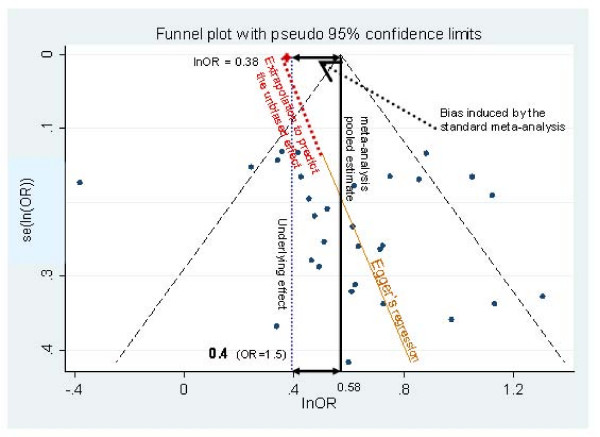
**Regression line and standard meta-analysis on a funnel plot of simulated asymmetrical data**.

Typically, the presence of small-study effects in meta-analysis is identified through asymmetry of a funnel plot. A funnel plot is a scatter plot of study effect size (usually on the x-axis) against a measure of study precision on the y-axis [5] (see Figure 1, ignoring the additional annotations). When no small-study effects are present, the funnel plot should resemble an inverted funnel with larger variation in effect sizes being observed in the less precise studies (due to sampling error). When small-study effects are present, the funnel will look asymmetrical with a tendency for effect sizes to be larger in the less precise studies, suggesting a missing 'chunk' out of the funnel on the left-hand side (presuming large positive effect sizes are beneficial).

Several statistical methods exist for detecting funnel plot asymmetry/small-study effects [6]. However, detection alone is i) limited since the likely impact of the bias is not assessed, [7]; ii) problematic since the chance of a false negative result are typically high; [8] and iii) insufficient if the results of the meta-analysis are to inform policy decisions. A reliable way to adjust meta-analysis for small-study effects is required to facilitate more reliable decision-making.

Disentangling the underlying cause of funnel plot asymmetry is difficult, although adding contours of statistical significance onto the plot has recently been suggested as a way of aiding interpretation [9]. However, whether the cause for funnel asymmetry is publication bias or other factors, predicting the effect in an infinitely large study – at the top of a funnel plot – can be perceived to be unbiased since i) if publication bias is the cause, larger studies will be less affected than smaller ones under the selection mechanisms assumed to underlie publication bias [10] (i.e. a hypothetical study of infinite size would have no chance of being suppressed and hence would provide an unbiased estimate of the population effect); and ii) if other confounding factors are the source of the small-study effects, the effect sizes of larger studies better represent the effects that would be seen when an intervention is implemented on a large scale, while smaller studies tend to be less representative of the population of interest. (Again, the effect size of interest is best represented by a hypothetical study of infinite size.)

We evaluate, through a comprehensive simulation study, a number of regression-based approaches to adjust a meta-analysis for publication bias. We also evaluate the performance of the models conditional on a statistically significant result from a test for publication bias. For comparison we also consider the Trim & Fill method [11], which is probably the most widely used adjustment method presently.

In the simulation study we have generated small-study effects by inducing PB using different models for study suppression. Therefore, for simplicity, we refer to PB as the cause of such small-study effects in the remainder of this paper. However, as argued above, we consider such an approach to be appropriate for all small-study effects in a decision making context.

The outline of this article is as follows. Section two presents a description of the statistical methods assessed. Section three describes the design of the simulation study. Section four presents the results of the simulation study, and Section five, the discussion, concludes the article.

## Methods

### Adjustment methods evaluated

The context considered throughout is that of 2-arm comparative studies reporting binary outcome data, with the meta-analysis being conducted on the (log) odds ratio scale. The different regression-based methods used to adjust for PB are described below. Additionally, for completeness we also consider the standard fixed and random effects meta-analysis models [12].

### Trim and fill

Trim & Fill [11,13] is probably the most popular method for examining the possible effect of PB on the pooled estimate, and can be defined as an iterative non-parametric adjustment method based on a rank-based data augmentation technique to account for asymmetry on the funnel plot. Briefly, the method "trims" the asymmetric studies on the right-hand side of the funnel for which there are no left-hand counterparts. A revised pooled estimate "adjusting for publication bias" is then derived from this reduced dataset. Then, the "trimmed" studies are reinstated and studies, assumed to be missing, are imputed on the opposite side of the funnel by "reflecting" the trimmed studies about the adjusted pooled effect line and uncertainty in the "adjusted" pooled effect is calculated using this augmented dataset.

We evaluate both L_0 _and R_0 _estimators [13] in the simulation study. However, since results were similar for both estimators relative to differences with the other methods, only the results from the R_0 _estimator are presented. We implement this method using fixed effects in both the 'trimming' and 'filling' parts of the algorithm (fixed-fixed), similarly, random effects for both parts (random-random), and fixed effects to 'trim' and random effects to 'fill' (fixed-random). Justification and evaluation of these variants is available elsewhere. [6,14]

### Regression methods

A number of regression models exist that assess the degree of association between the study effect and a measure of its precision. The initial test based on the statistical significance of this association was suggested by Egger *et al*. [10], but due to concerns regarding its performance on the odds ratio scale, several modifications have now been proposed and are also considered [15,16]. A further arcsine-based regression test developed by Rücker et al. [17] is not considered further here because it performs a correction on the arcsine scale, which is harder to directly compare with the other methods performance.

Evidence of such an association may suggest that the meta-analysis is affected by PB(/small-study effects) if the observed smaller, less precise, studies have larger effect sizes than the more precise studies [4,18]. This association can be illustrated by the regression line on a funnel plot (figure 1). This figure presents a standard funnel plot [19] of outcome (ln(OR)) against a measure of study precision (se(lnOR)), for a simulated dataset with an underlying ln(OR) of 0.4 but with PB induced (i.e. studies are suppressed in bottom left hand side of plot). This regression line indicates how the less precise studies tend to have, on average, larger treatment effects, implying that PB(/small study effects) bias exist.

By extrapolating this regression line to the point where the standard error is zero, the effect size from a (hypothetical) infinitely large study can be predicted. And, as discussed in Section 1, this can be interpreted as the effect size adjusted for PB(/small-study effects). For example in Figure 1, a standard fixed effects meta-analysis estimates a pooled effect size of ln(OR) = 0.58, which is considerably larger than the true underlying effect (lnOR = 0.4). When meta-regression is applied to the dataset, where the independent variable is se(ln(OR)), this predicts a ln(OR) of 0.38 for a se(ln(OR)) of 0, which is closer to the underlying truth. The notion of adjusting for PB through incorporation of study precision as the meta-regression covariate has been suggested previously [4,20-22] but not formally evaluated. Details of these various regression methods applied in this way are described below.

**'Egger' ***et al*. [10] proposed a regression test for funnel asymmetry that is widely used. The original equation published by Egger et al. [10] can be shown to be equivalent to a weighted linear ordinary least squares regression model with standard error as a covariate [4]:

(1a) $$\begin{array}{cccc} y_{i}=\alpha +\beta \times se_{i}+\varepsilon_{i} & \text{weighted by }\frac{1}{se_{i}^{2}} & \text{with} & \varepsilon_{\text{i}}\sim N(0,se_{i}^{2}\times \phi ) \end{array}$$

where *y*_*i *_is the ln(OR) from study *i *(in the context considered in this paper) and *se*_*i *_is its associated standard error. We interpret the two coefficients *α *and *β *to represent the adjusted pooled effect (intercept) and the slope associated with funnel plot asymmetry respectively. The regression is weighted by the inverse of the estimated effect size variance for each study (1/*se*_*i *_^2^). *φ *is an unknown multiplicative dispersion parameter that is estimated in the regression and allows for possible heteroscedasticity [23,24].

The notion of this multiplicative error term in this context is not consistent with the typically used variance-weighted meta-regression models where the error is additive [23]. As a result, we also implement an alternative model with an additive error i.e.

(1b) $$\begin{array}{cccc} y_{i}=\alpha +\beta \times se_{i}+\varepsilon_{i} & \text{weighted by }\frac{1}{se_{i}^{2}} & \text{with} & \varepsilon_{i}\sim N(0,se_{i}^{2}) \end{array}$$

This fixed effects regression model assumes homogeneous effect sizes. While the multiplicative dispersion parameter *φ *in equation (1.a) allows for the incorporation of between-study variability [23], the more usual way of allowing for heterogeneity in a meta-analysis is through the incorporation of a random effect into the model. In this way, the between-study variance is modelled, not as a multiplicative component (*φ*), but, as an additive component of between-study variance (*τ*^2^) to the within-study variance (*se*_*i *_^2^):

(1c) $$\begin{array}{l} \begin{array}{cc} y_{i}=\alpha +\beta \times se_{i}+\mu_{i}+\varepsilon_{i} & \text{weighted by }\frac{1}{se_{i}^{2}+\tau^{2}} \end{array}\\ \begin{array}{cccc} \text{where} & \mu_{i}\sim N(0,\tau^{2}) & \& & \varepsilon_{i}\sim N(0,se_{i}^{2}) \end{array}. \end{array}$$

Here, *μ*_*i *_is a normal error term with mean zero and variance *τ*^2 ^to be estimated from the data.

**'Egger-Var**' methods are modifications of the Egger model variants (equations 1.a-c) created by replacing the standard error of each study's effect size with the corresponding variance as the predictor variable. This implies that the relationship between effect size and its variance is linear, whereas the Egger approach assumes linearity in relation to the standard error.

(2a) $$\begin{array}{cccc} y_{i}=\alpha +\beta \times se_{i}^{2}+\varepsilon_{i} & \text{weighted by }\frac{1}{se_{i}^{2}} & \text{with} & \varepsilon_{\text{i}}\sim N(0,se_{i}^{2}\times \phi ) \end{array}$$

(2b) $$\begin{array}{cccc} y_{i}=\alpha +\beta \times se_{i}^{2}+\varepsilon_{i} & \text{weighted by }\frac{1}{se_{i}^{2}} & \text{with} & \varepsilon_{i}\sim N(0,se_{i}^{2}) \end{array}$$

(2c) $$\begin{array}{l} \begin{array}{cc} y_{i}=\alpha +\beta \times se_{i}^{2}+\mu_{i}+\varepsilon_{i} & \text{weighted by }\frac{1}{se_{i}^{2}+\tau^{2}} \end{array}\\ \begin{array}{cccc} \text{where} & \mu_{i}\sim N(0,\tau^{2}) & \& & \varepsilon_{i}\sim N(0,se_{i}^{2}) \end{array} \end{array}$$

**'Harbord' **is a regression test for small-study effects [16]. A distinct advantage of this method is that it reduces the correlation between the ln(OR) and its corresponding standard error which causes asymmetry in funnels even when no small-study effects exist [25]. The statistical model can be expressed as:

(3) $$\begin{array}{l} \begin{array}{cccc} \frac{Z_{i}}{V_{i}}=\beta +\frac{\alpha }{\sqrt{V_{i}}}+\omega_{i} & \text{weighted by }V_{i} & \text{where} & \omega_{i}=\frac{\varepsilon_{i}}{\sqrt{V_{i}}} \end{array}\\ \begin{array}{ccc} \mathrm{var}\left(\omega_{i}\right)=\frac{\mathrm{var}\left(\varepsilon_{i}\right)}{V_{i}}=\frac{\sigma^{2}}{V_{i}} & \text{and thus} & \omega_{i}\sim N\;\left(0,\frac{\sigma^{2}}{V_{i}}\times \phi \right) \end{array} \end{array}$$

where *Z*_*i *_is the efficient score and *V*_*i *_is Fisher's information (the variance of *Z *under the null hypothesis for the *i*^*th *^study).

**'Peters**' corresponds to another regression test for funnel asymmetry [15]. This weighted linear ordinary least squares regression model establishes a linear association between-study effect and its sample size weighted by a function of sample size. Peters *et al.*'s approach is a modification of Macaskill's test [25] which it outperformed under simulation [15], with the inverse of the total sample size as the independent variable. Using a function of the sample size as the predictor variable avoids the structural dependence between ln(OR) and var(ln(OR)) (N.B. the problem which also motivated the development of the Harbord method). It also avoids violating an assumption of regression analysis infringed by all the previous methods, which is that the covariates are estimated but error is ignored [26].

(4) $$\begin{array}{l} \begin{array}{cccc} y_{i}=\alpha +\frac{\beta }{a_{i}+b_{i}+c_{i}+d_{i}}+\varepsilon_{i} & \text{weighted by} & {\left(\frac{1}{a_{i}+b_{i}}+\frac{1}{c_{i}+d_{i}}\right)}^{-1} & \text{where} \end{array}\\ \varepsilon_{\text{i}}\sim N(0,se_{i}^{2}\times \phi ) \end{array}$$

As before, the two coefficients *α *and *β *represent the adjusted pooled effect (intercept) and the regression slope respectively. For each study *i*, *a *and *b *represent the observed number who experience the outcome of interest in the treated and control groups, respectively, and *c *and *d *are the numbers corresponding to those not developing the outcome in the treated and control group respectively. Thus, the sample size of the ***i***^*th *^study corresponds to the sum of *a*_*i*_*, b*_*i*_*, c*_*i *_and *d*_*i*_.

### Conditional methods

We suspect, in practice, researchers carry out a test for small-study effects and consider the use of adjustment methods conditional on the outcome of such a test. Therefore, we also evaluate two conditional approaches in which a standard random effects model or either of the original Egger or Harbord adjustment based methods are used depending on whether the corresponding test (i.e. Egger or Harbord respectively) was significant at the 10% level. Since the Egger conditional approach is almost always outperformed by the Harbord conditional method, only the latter is reported below.

### Summary of adjustment methods

In summary, the performance of the following adjustment methods is reported below. An abbreviation is given to each method, which is used in the remainder of the paper:

• The two usual standard meta-analysis methods

• Fixed effects meta-analysis (*FE*)

•; Random effects meta-analysis (*RE*)

• Non-parametric adjustment method: Trim & Fill

• R_0 _estimator, trim using fixed effects & fixed effects on filled dataset (*TF FE-FE*)

• R_0 _estimator, trim using fixed effects & random effects on filled dataset (*TF FE-RE*)

• R_0 _estimator, trim using random effects & random effects on filled dataset (*TF RE-RE*)

• Parametric adjustment methods: weighted regressions

• Egger's model variants:

• Fixed effects *(FE-se)*;

• Random effects *(RE-se)*

• Dispersion *(D-se)*

• Egger-Var model variants:

• Fixed effects *(FE-var)*

• Random effects *(RE-var)*

• Dispersion *(D-var)*

• Other regressions

• Harbord's model *(Harbord)*

• Peters' model *(Peters)*

• Conditional method

• PB test plus conditional adjustment based on it using Harbord's model (*Harbord-C*)

### Simulation Study

#### Methods for generating the meta-analysis datasets

Simulated meta-analyses were based on a set of characteristics intended to reflect meta-analyses of randomised clinical trials in the medical literature. The assumptions made and parameter values chosen have drawn on the authors' extensive experience in this area as well as considering the complete review of previous simulation studies in the field [27].

Scenarios in which 5, 10, 20 or 30 individual trials were included in the meta-analysis were explored [4]. The sample size of the individual studies within each meta-analysis was generated from a log normal distribution with mean 6 and variance 0.6. This reflects the greater number of small studies compared to large studies as commonly observed in real meta-analyses. This distribution results in a mean (median) size of 483 (403) individuals per study and a standard deviation of 318. The 1% (99%) percentile is 100 (1628) individuals per study. The numbers of individuals allocated to treatment and control arms was equal for all simulations.

Both fixed (homogeneous) and random effects (heterogeneous) meta-analysis scenarios were simulated. Underlying effect sizes (i.e. for all studies under fixed effects and for the mean of the distribution of studies under random effects) considered were OR = 1 (null effect), 1.5 and 3 (representing a large effect), where OR > 1 is considered clinically beneficial. Following the approach of Schwarzer's *et al*. [28], the event rate for the intervention and control arms is modelled by simulating the average event probability of the treatment and control trial arms. (This approach reduces the correlation between individual effect estimates and their corresponding standard errors compared to modelling the event rate on the control group). The average event probability for each trial was generated according to a uniform distribution (0.3, 0.7). The actual number of events in each study arm was generated according to a binomial distribution taking into account the corresponding arm event probability and study arm size.

To simulate each trial within each meta-analysis under fixed effects, the following model was used:

(5) $$\begin{array}{c} r_{i}^{C}\sim \text{Binomial}(p_{i}^{C},n_{i}^{C})\\ r_{i}^{T}\sim \text{Binomial}(p_{i}^{T},n_{i}^{T})\\ \text{logit}(p_{i}^{C})=\mu_{i}-\delta /2\\ \text{logit}(p_{i}^{T})=\mu_{i}+\delta /2 \end{array}$$

Where underlying ln(OR) is *δ*, and *μ*_*i *_is the average event rate on the logit scale in the *i*^*th *^study. *n*_*i*_*, p*_*i*_*r*_*i *_are the number of subjects, the probability of an event (derived directly from *μ*_*i *_and *δ*), and the number of events in the *i*^*th *^study arm respectively, with a superscript *C *or *T *indicating the control or treatment group.

Between-study variability (heterogeneity) is simulated here since it has been reported to be an important variable in determining the performance of methods to adjust for PB [29,30]). Data were generated according to the model below [31].

(6) $$\begin{array}{c} r_{i}^{C}\sim \text{Binomial}(p_{i}^{C},n_{i}^{C})\\ r_{i}^{T}\sim \text{Binomial}(p_{i}^{T},n_{i}^{T})\\ \text{logit}(p_{i}^{C})=\mu_{i}-\delta_{i}/2\\ \text{logit}(p_{i}^{T})=\mu_{i}+\delta_{i}/2\\ \delta_{i}\sim \text{N}(\theta ,\tau^{2}) \end{array}$$

Now the underlying effect in the *i*^*th *^trial, *δ*_*i *_is assumed to be drawn from a Normal distribution with mean value *θ *and between-study variance *τ*^2^. *τ*^2 ^is defined to be either 0%, 100%, 150% or 200% of the average within-study variance for studies from the corresponding fixed effects meta-analyses simulated. [15]

The between-study variance can also be defined in terms of *I*^2 ^(the percentage of total variation across studies that is due to between-study variation rather than sampling error [32]). In a scenario where PB is absent, a between-study variation of 0% & 150% of the average within-study variation corresponds to an average *I*^2 ^of 7% & 57% respectively. Note that under scenarios where PB is simulated, this will affect estimates of the between-study variability [33].

The two most commonly assumed selection processes used in previous simulation studies to induce PB are considered here:

#### 1) Publication suppression on the basis of a one-sided p-value associated with the effect estimate of interest [14,25,33-36]

The probability that a study is published depending on its resulting p-value is modelled by a step function with discontinuities determined by two cut-points as shown in Table 1. Following Peters' [37] and Hedges' [34] approach, two levels of bias were induced to represent "moderate" and "severe" PB (Table 1); for example, under severe PB, only 25% of studies with a p-value larger than 0.2 are published and subsequently included in the meta-analysis.

**Table 1:** Specification of publication bias severity based on one-sided significance

Severity of Publication Bias	p-value from study	Probability study included in MA
Moderate	< 0.05	1
	0.05 – 0.5	0.75
	> 0.5	0.25

Severe	< 0.05	1
	0.05 – 0.2	0.75
	> 0.2	0.25

#### 2) Suppressing the most extreme unfavourable results

This assumes that only the estimated effect size influences whether a study is included in the meta-analysis or not, so that studies with the most extreme unfavourable estimates of effect are excluded [11,14]. Here, the number of studies excluded does not depend on the underlying effect size, as it does when PB is induced on the basis of p-value. "Moderate" and "severe" PB were represented by excluding either the 14% or 30% of the most extreme studies showing an unfavourable effect such that the final number of studies in a meta-analysis was reached. For example, under severe level of PB, where the meta-analysis size ought to be 30, 50 studies are generated so that 20 studies (i.e. 30% of the original 50 studies) giving the most extreme unfavourable estimates are omitted from the meta-analysis.

### Simulation scenarios investigated

**Figure 2:**
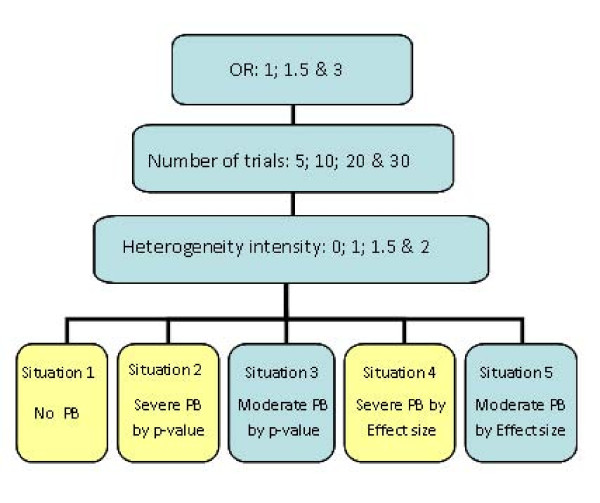
**Schematic outlining the meta-analysis scenarios simulated**. 5000 meta-analysis datasets for each combination of the 3 underlying odds ratios, 4 different numbers of trials in the meta-analysis, 4 levels of heterogeneity, and 5 PB situations (240 scenarios in total) were generated.

The meta-analytic scenarios used to investigate the performance of the alternative adjustment methods are intended to encompass a comprehensive collection of realistic situations. Figure 2 outlines the scenarios considered.

Altogether there are 5 different PB situations (none, and moderate and severe for both p-value and effect size suppression). Each of these situations is applied to all combinations of the following meta-analysis characteristics: underlying effect size (OR = 1, 1.5 & 3), number of studies in the meta-analysis (5, 10, 20 & 30) and degree of between-study heterogeneity (0%, 100%, 150% or 200% of the average within-study variance). This results in 240 individual scenarios, with 5,000 simulated datasets generated for each scenario. All methods summarised in section 2.4 were applied to each of these datasets. All statistical analyses were performed using Stata, version 9.2 [38].

Results of this simulation study are reported in section 4; although not all scenarios are shown due to the negligible added value they provide to the overall conclusions. In particular, scenarios with "moderate" bias are omitted because they follow the same overall trend as the more severe scenarios, but with differences between methods tending to be less pronounced. Hence the PB situations labelled 1, 2 and 4 in Figure 2 (none and severe by both mechanisms) are reported here. (For completeness, a web-only appendix containing the remaining scenarios are available from [see Additional file 1]).

### Criteria to assess the methods performance

In order to evaluate the adjustment methods, their performance is assessed through measures of model accuracy (bias), precision (variance) and coverage (type I error rate) [39] in the simulation study.

We consider *"absolute bias*" – the expected difference between the estimated (adjusted) effect and the underlying true effect across all simulations. A negative bias indicates an under-estimate of the true underlying effect, and a positive residual bias indicates an over-estimate of the true underlying effect. Arguably, when interpreting absolute bias, it is important to consider how close the true effect is from the null effect. It is desirable for the adjustment methods to work well near the null where small changes may have impact on the direction of the effect and hence any conclusions. Conversely, if the performance is poorer further away from the null effect (e.g. OR = 3), the consequences are, in some respects, less of a concern because it is unlikely that PB will change the direction of the pooled effect.

In addition, we consider the mean squared error (*MSE*) which incorporates both the variance and bias of the estimator [39]:

(7) $$\text{MSE }(\hat{\theta })=\frac{\Sigma \left({\left({\hat{\theta }}_{j}-\theta \right)}^{2}\right)}{N}$$

Where ${\hat{\theta }}_{j}$ is the estimated ln(OR) predicted by the model for the *j*^th ^simulated meta-analysis (*j:1, ..., N*; where *N *is the total number of simulations). The underlying true value of ${\hat{\theta }}_{j}$ is *θ*. The MSE can be thought of as corresponding to the sum of the variance plus the square of bias of $\hat{\theta }$.

We also consider the *coverage probability*, which can be defined as the proportion of simulations in which the true underlying effect lies within the 95% confidence interval of the predicted effects simulated. This informs how well the type I error is controlled by the statistical model. The coverage probabilities should be approximately equal to the nominal coverage rate to properly control the type I error rate for testing a null hypothesis of no difference in effect size between the true underlying effect and the predicted one; the 5% level is used throughout.

The final measure considered is the average "*variance*" of the predicted pooled effects. This is used to ascertain the contribution of bias and variance to the MSE.

## Results

**Figure 3:**
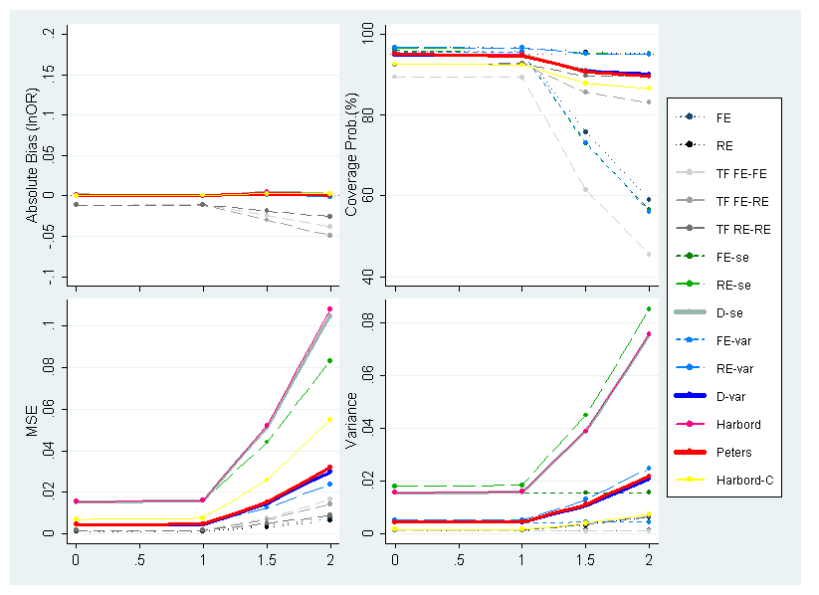
**Measures of absolute bias, coverage probabilities, MSE and precision of the predicted effect for meta-analyses simulated to have 30 studies, an underlying OR of 1 (lnOR = 0) and no PB alongside increasing levels of heterogeneity (PB situation 1)**.

**Figure 4:**
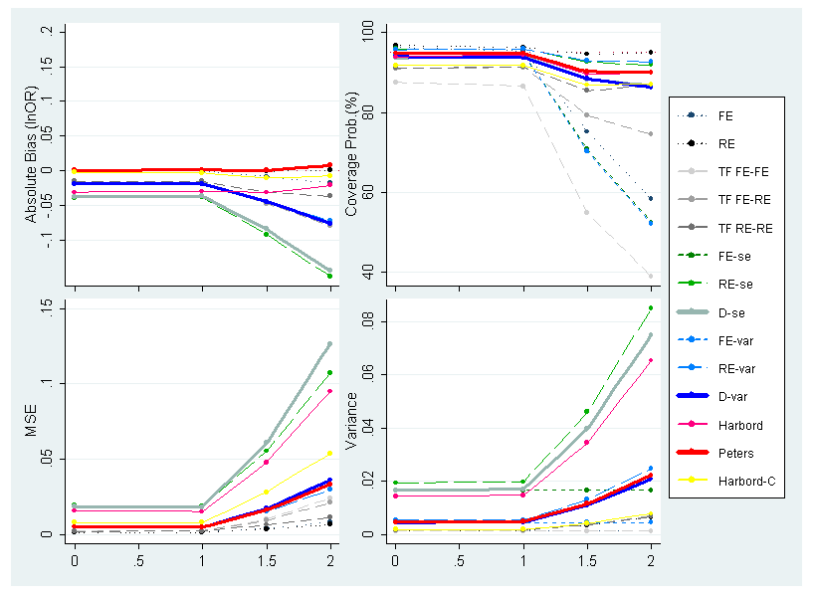
**Measures of absolute bias, coverage probabilities, MSE and variance of the predicted effect for meta-analyses simulated to have 30 studies, an underlying OR of 3 (lnOR = 1.1) and no PB alongside increasing levels of heterogeneity (PB situation 1)**.

Figures 3 and 4 present the results of simulated meta-analyses of 30 studies with no PB and underlying OR = 1 and OR = 3 respectively while varying the degree of between-study heterogeneity (defined on the x-axis of all plots). Unsurprisingly, when PB is not induced, the standard random-effects meta-analysis estimator performs best and provides an unbiased estimate, correct 95% coverage probability and the lowest MSE of all methods examined. When the underlying OR = 1, the majority of the other methods also perform very well (figure 3), although the overall performance is significantly reduced once the between-study variance exceeds the within-study variances; i.e. *I*^2 ^> 50%. Generally, the methods which allow for heterogeneous data (i.e. include random effects or dispersion parameters) obtain more reasonable coverage probabilities than those which do not.

When the underlying effect is increased to OR = 3 (figure 4), greater variability in results of the different methods is observed, with generally worse performance seen for most methods. This is due, at least in part, to the susceptibility of some methods to the induced artefactual relationship between ln(OR) and its se(lnOR) which increases as the OR increases. Indeed, while the standard random effect model has no bias, the two adjustment methods with lowest absolute bias are those using the Peters and the conditional Harbord models which were developed to circumvent the problems of the artefactual relationship. Note also that the Harbord, Egger RE (RE-se) and dispersion (D-se) methods report large MSE values. This can be partially explained by their large variance in the model predictor. With respect to the Egger based methods, the use of the variance as the predictor variable provides less biased and more precise estimates than using the standard error.

**Figure 5:**
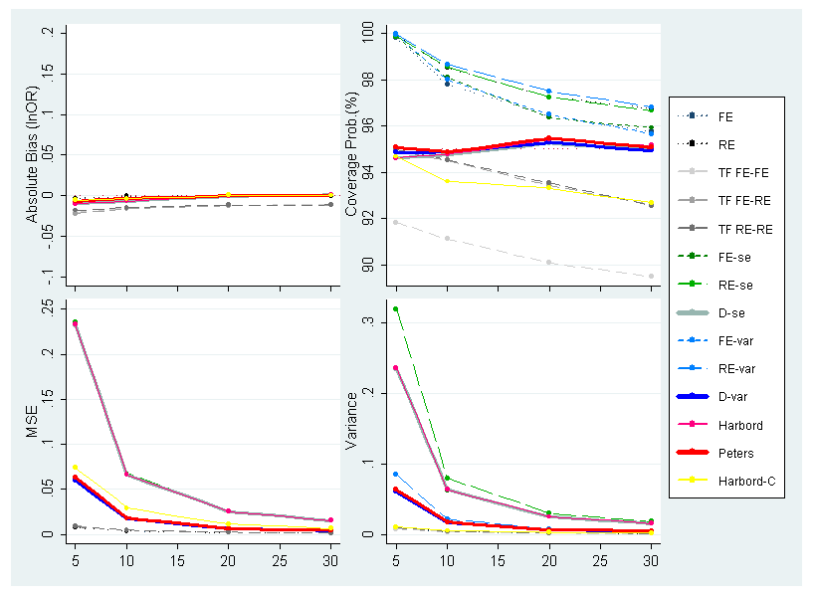
**Measures of absolute bias, coverage probabilities, MSE and precision of the predicted effect for homogeneous meta-analyses and underlying OR of 1 (lnOR = 0) and no PB alongside increasing meta-analysis sizes (PB situation 1)**.

Figure 5 presents results of simulated meta-analysis with an underlying OR of 1, where no PB is induced and *τ*^2 ^= 0, while varying the number of studies included in the meta-analysis. Figure 5 reveals how several methods, i.e. both FE and RE standard meta-analyses and FE & RE Egger-based methods (FE-se, RE-se, FE-var & RE-var), report substantial overcoverage values – well above the pre-specified 95% – particularly for meta-analyses including less than 30 studies. Whereas the methods which include a dispersion parameter retain more appropriate coverage probabilities. Nevertheless, the overcoverage problem disappears under heterogeneous conditions (not shown); implying that the methods listed above are unable to provide reasonable coverage probabilities for fairly homogeneous data (i.e. approximately *I*^2 ^< 10%) for meta-analyses with typically small numbers of studies (i.e. below 30 studies).

**Figure 6:**
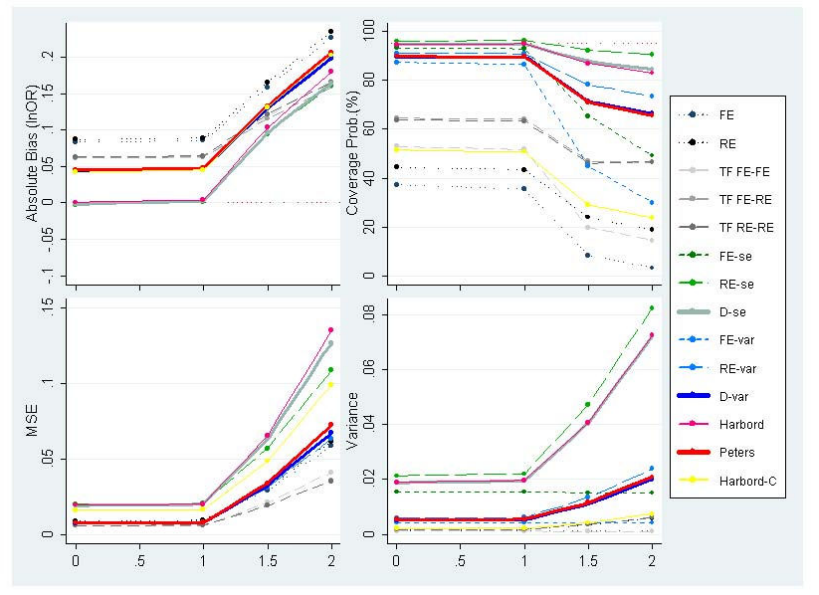
**Measures of absolute bias, coverage probabilities, MSE and variance of the predicted effect for meta-analyses simulated to have 30 studies, an underlying OR of 1 (lnOR = 0) and severe PB induced by p-value alongside increasing heterogeneity (PB situation 2)**.

**Figure 7:**
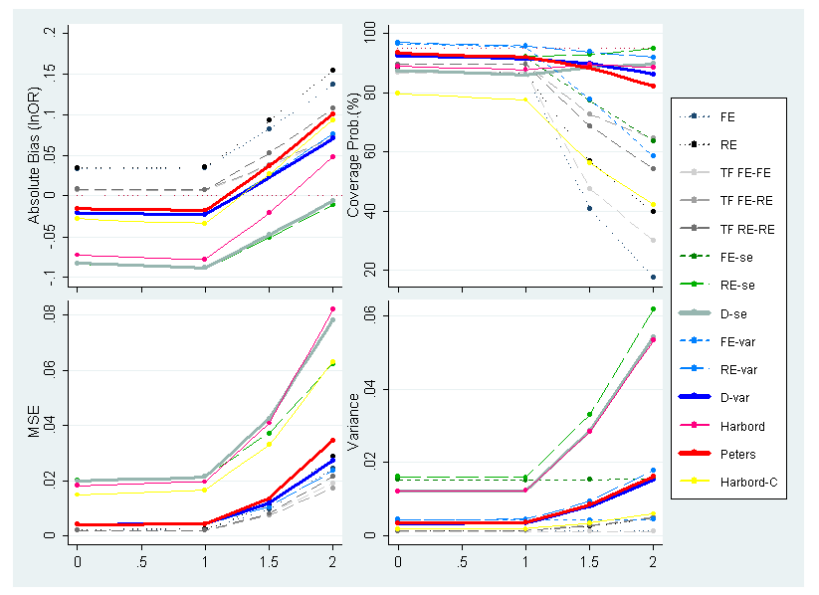
**Measures of absolute bias, coverage probabilities, MSE and variance of the predicted effect for meta-analyses simulated to have 30 studies, an underlying OR = 1.5(lnOR = 0.4) and severe PB induced by p-value alongside increasing heterogeneity (PB situation 2)**.

Figures 6 and 7 display the results of simulations with severe PB induced by one-sided p-values for OR = 1 & 1.5 respectively. In these plots the degree of between-study heterogeneity is varied along the x-axis. OR = 1.5 is reported rather than OR = 3 because, in the latter case very few studies would be suppressed under this selection mechanism as the majority of studies are highly statistically significant (i.e. similar results are obtained to those presented previously in figure 4). When severe PB is induced, standard meta-analysis methods, and to less extent all Trim & Fill variants, do not perform well, producing biased estimates and poor coverage probabilities. Figures 6 and 7 also suggest that none of the adjustment methods perform particularly well under large values of heterogeneity combined with extreme PB.

The degree of absolute bias is dependent on the underlying odds ratio. This can be explained by p-value induced PB causing 'disfigurement' to the funnel plot which is dependent on the underlying odds ratio; i.e. the funnel plot is almost intact under OR = 3, while a very asymmetrical shape is obtained under OR = 1. The methods that can accommodate heterogeneous data (through the inclusion of random effects or dispersion parameters) are the ones with the most appropriate coverage probabilities among the ones evaluated; these include the Harbord, Peters as well as the Egger-based methods (RE-se, D-se, RE-var & D-var). However, the Harbord and the two Egger (RE-se & D-se) methods report substantially inflated MSE and variance values compared to other methods evaluated.

**Figure 8:**
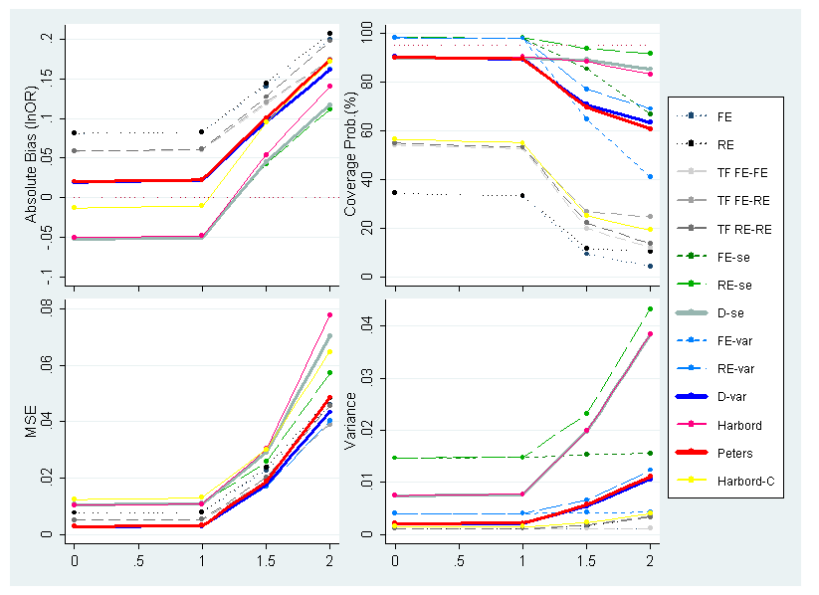
**Measures of absolute bias, coverage probabilities, MSE and variance of the predicted effect for meta-analyses simulated to have 30 studies, an underlying OR = 1 (lnOR = 0) and severe PB induced by effect size alongside increasing heterogeneity (PB situation 4)**.

In Figure 8, we consider severe PB induced by effect size on meta-analyses of 30 studies with an underlying OR of 1 while varying the amount of between-study heterogeneity. Since inducing PB in this manner has the same effect on the funnel shape regardless of the underlying effect size simulated (unlike suppression based on one-sided p-value), only OR = 1 is exhibited here. Nevertheless, the performance of some of the methods will still be dependent to some degree on the underlying effect size due to the induced correlation mentioned earlier (not shown but available in the web-only appendix [see Additional file 1]).

The conditional regression method, Trim & Fill and standard meta-analysis estimators do not perform particularly well due to low coverage and large amounts of residual bias. As before, for larger values of heterogeneity, the Egger-Var methods (FE-var, RE-var & D-var) tend to report somewhat lower MSE and coverage probabilities than the Egger ones (FE-se, RE-se & D-se) thanks to their restrained variance.

Also of note is that, under fairly homogeneous effects, adjustment methods based on FE (FE-se & FE-var) and RE (RE-se & RE-var) Egger models provide coverage probabilities well above 95%, implying inappropriately small type I error rates. This can be explained by the inability of these models to accommodate the underdispersion of observed effects (i.e. less variability than would be expected by chance) caused by PB. Conversely, the methods which include a dispersion parameter do not suffer from excessive coverage probabilities due to the fact that they can accommodate underdispersion by allowing the dispersion parameter below the value of one. This means that the Harbord, Peters and the Egger methods (D-se & D-var) perform favourably under scenarios where PB causes underdispersion.

## Discussion

In this paper we have compared some novel and existing methods for adjusting for publication bias through an extensive simulation study. Results are encouraging, with several of the regression methods displaying good performance profiles. Overall, no particular method consistently outperforms all others. The overall performance of all the methods deteriorates as *I*^2 ^exceeds 50% [30] and the underlying odds ratio increase; while at the same time differences between them diverge.

With respect to the popular Trim & Fill method, we find it hard to recommend over the regression-based alternatives due to its potentially misleading adjustments and poor coverage probabilities, especially when between-study variance is present [14,29], although it should be acknowledged that Trim & Fill was only intended as a form of sensitivity analysis [11] rather than as an adjustment method per se.

Although the standard meta-analysis models are a good approach under lack of PB, they inevitably perform poorly when PB is present. This motivated the examination of regression-based adjustment methods conditional on their associated test for PB. Such an approach is also of interest because it may reflect what is commonly done in practice when dealing with suspected PB. Unfortunately, these conditional approaches did not perform as well as the (unconditional) alternatives. This may be explained by the fact that all existing tests for PB suffer from low statistical power [4,7,25] leading to inappropriate methods being used in some instances, and, this is a warning to not using such an approach (formal or informally). This is an inherent problem of pre-tests since the failure of the pre-test to reject the null-hypothesis does not prove the null hypothesis true, unless the pre-test was designed as an equivalence test.

The persistent low level of coverage probability by the fixed effects Egger models (FE-se & FE-var) under heterogeneous settings render them inappropriate. Equally, coverage probabilities above the 95% threshold produce inaccuracy on the confidence interval; which potentially biases any subsequent assessment of uncertainty around the estimate of interest. This is a serious concern in a decision-making context, where alternative treatments may report similar mean effect sizes. In such cases, accurate quantification of uncertainty to allow discriminating among treatments is vital to facilitate realistic probabilistic statements about, say, cost-effectiveness relative to the alternative treatments. Here, both fixed effects (FE-se & FE-var) and random effects (RE-se & RE-var) Egger models tend to suffer from excessive coverage probabilities under:

• Scenarios of underdispersion caused by severe PB (figure 8);

• Mostly homogeneous settings (figures 5, 7, 8), provided the meta-analysis is not exceptionally large (i.e. less than 30 studies); and

• Small size meta-analysis (figure 5), provided the data is fairly homogeneous.

Additionally, since in practice it will often be difficult to determine whether heterogeneity is present or not (due to the low power of associated test and distortions caused by PB) this makes appropriate implementation of fixed effect methods difficult.

Over the range of simulation scenarios considered, the Harbord, Peters and both Egger dispersion (D-se & D-var) methods would appear to have best overall performance. They do not always produce the least biased estimate, but they do consistently retain good coverage probability levels (by equally accommodating homogeneous and heterogeneous data), while keeping competitive with respect to bias.

However, when faced with small size meta-analyses and/or heterogeneity (figures 3, 4, 5, 6, 7, 8), the outstanding coverage comes to a high cost in terms of MSE for the Harbord and Egger dispersion (D-se) methods compared to the other two. These two methods tend to report low residual bias but yet persistent high MSE values, due to the large variances. In contrast, Peters and the Egger-var (D-var) methods report slightly lower coverage probabilities besides much lower MSE values as a result of their restrained variance. Due to this, we recommend the Peters and Egger-var (D-var) methods which perform very similarly throughout the simulations: at least in terms of coverage, MSE and variance. However, there is one instance (figure 4) where they clearly differ with regard to absolute bias; which can be explained by the Peters' method profiting from avoiding the structural correlation problem between outcome and standard error by using a function of sample size as the predictor variable.

One favourable factor in this simulation study is that there was always considerable variation in the sizes of the studies in each dataset. Again, the methods performance will deteriorate if studies sizes are less variable. This is particularly a concern for the regression approaches if all the studies are small, since a larger extrapolation to the intercept would be required.

In these simulations we defined levels of heterogeneity in terms if *I*^2 ^(the percentage of total variation across studies that is due to between-study variation rather than sampling error). By doing this, heterogeneity is induced proportionally to the within-study variation. By defining heterogeneity in terms of the *I*^2 ^statistic means we are focussing on the impact rather than the extent of heterogeneity [32] across the different meta-analytic scenarios. An alternative modelling approach would be to define heterogeneity in terms of the between-study variance parameter (*τ*^2^) which would lead to an assessment of the methods with respect to absolute degrees of between-study variability. Previous studies that evaluate publication bias methods [15-17,28] have used a mixture of these approaches and it is not clear which, if either, is superior.

Other methods for PB adjustment are available but were not evaluated in the simulation study. These include a literature on the use of selection modelling techniques [40]. The reason for excluding them is twofold: 1) Unless there are large numbers of studies, it will be necessary to specify the selection mechanism as a modelling assumption. Hence their performance will directly depend on how good the specification of the selection model is and this is difficult to evaluate via simulation (i.e. if you specify the selection model to be the same as used to simulate the data you can guarantee good performance and vice versa). 2) Previous work has acknowledged that since the selection mechanism is not identifiable from the data, sensitivity analyses should be carried out using a range of selection functions. While this is potentially useful in an inference making context where robustness or lack of it may be explored over a range of possible selection models, it is less useful in a decision making context where a single decision has to be made.

Recently Copas and Malley [22] presented a novel way of obtaining a robust p-value for effect in a meta-analysis with publication bias based on a permutation test. Interestingly, this is shown to be closely related to the correlation found in the associated radial plot, which in turn is closely related to a funnel-plot related regression [4].

Since in medical applications any PB selection mechanisms will be unknown and there will often be too few studies to estimate it from the data, we believe regression-based methods, which make no explicit assumptions about the underlying selection mechanism, may have a useful role in a decision-based context.

We believe that a broad range of plausible meta-analyses situations have been evaluated through the scenarios evaluated in the simulation study. And that, given the variability and limited scope of some of the previous simulation studies in the evaluation of methods to address PB in the past, it would be desirable for there to be a consensus simulation framework in which future tests and adjustment methods could be evaluated. To this end, the comprehensive framework developed here could form the starting point for future simulation studies.

## Conclusion

In conclusion, several regression-based models for PB adjustment performed better than either the Trim & Fill or conditional (regression-based) approaches. Overall, the Egger-var (D-var) and Peters methods are identified as methods with potentially appealing statistical properties for PB adjustment with the Peters method performing better for large odds ratios under the simulation scenarios evaluated. However, it should be acknowledged that while our simulations were extensive, differences in results may be observed if different simulation parameter values were used.

Further research is considered worthwhile given our encouraging initial results. To this end, further work exploring the incorporation of information obtained from external sources to form a prior distribution for the regression coefficients is being developed in the hope of improving performance of the regression-based methods.

Finally, while we acknowledge that while publication bias is a problem that will not entirely disappear regardless of the statistical method of analysis, ignoring it is an unwise option [41]. We also support prevention of PB as a more desirable approach compared to detection or adjustment [8,16,42]. However, despite the limitations of existing methods we believe it is helpful to attempt to adjust for PB as long as it is present in the literature, particularly in a decision making context [34].

## Competing interests

The authors declare that they have no competing interests. The first author was supported by a Medical Research Council (MRC) Health Services Research Collaboration (HSRC) Studentship. The funding agreement ensured the authors' independence in designing the study, interpreting the data, writing, and publishing the report.

## Authors' contributions

AJS and AEA conceived the project and guided SGM in conducting the project. AJS, JLP & SGM designed the simulation study and carried out the statistical analyses. TDS, AEA, KRA & NJC participated in data analysis and interpretation. SGM designed and developed the plots. SGM & AJS drafted the paper, which was later revised by all co-authors through substantial contributions to the contents of the paper. All authors read and approved the final version of the manuscript for publication.

## Pre-publication history

The pre-publication history for this paper can be accessed here:

http://www.biomedcentral.com/1471-2288/9/2/prepub

## Supplementary Material

Additional file 1 **Additional results from the simulation study.** Additional plots summarising the simulation results for the remaining scenarios. Click here for file
