# Supplementary material for: Assessment of regression-based methods to adjust for publication bias through a comprehensive simulation study
Source: BMC Med Res Methodol. 2009 Jan 12;9:2. doi: 10.1186/1471-2288-9-2 (PMC2649158; doi:10.1186/1471-2288-9-2)
Supplement: Additional file 1 — Additional results from the simulation study. Additional plots summarising the simulation results for the remaining scenarios. [file 1471-2288-9-2-S1.doc]

**Appendix:** Additional plots summarising the simulation results from the remaining scenarios, where homogeneous and heterogeneous meta-analysis refers to a heterogeneity factor of 0 and 1.5 respectively.

**Publication bias situation 1**

**Figure A1** Measures of absolute bias, coverage probabilities, MSE and precision of the predicted effect for meta-analyses simulated to have 30 studies, an underlying OR of 1.5 and no PB alongside increasing levels of heterogeneity

**Figure A2** Measures of performance of the predicted effect for homogeneous meta-analyses and underlying OR of 3 and no PB alongside increasing meta-analysis sizes

**Figure A3** Measures of performance of the predicted effect for heterogeneous meta-analyses and underlying OR of 3 and no PB alongside increasing meta-analysis sizes

**Publication bias situation 2**

**Figure A4** Measures of performance of the predicted effect for homogeneous meta-analyses and underlying OR=1 and severe PB induced by p-value alongside meta-analysis sizes

**Figure A5** Measures of performance of the predicted effect for heterogeneous meta-analyses and underlying OR=1 and severe PB induced by p-value alongside meta-analysis sizes

**Figure A6** Measures of performance of the predicted effect for homogeneous meta-analyses and underlying OR=1.5 and severe PB induced by p-value alongside meta-analysis sizes

**Figure A7** Measures of performance of the predicted effect for heterogeneous meta-analyses and underlying OR=1.5 and severe PB induced by p-value alongside meta-analysis sizes

**Publication bias situation 3**

**Figure A9** Measures of performance of the predicted effect for homogeneous meta-analyses and underlying OR=1 and moderate PB induced by p-value alongside meta-analysis sizes

**Figure A10** Measures of performance of the predicted effect for heterogeneous meta-analyses and underlying OR=1 and moderate PB induced by p-value alongside meta-analysis sizes

**Figure A11** Measures of performance of the predicted effect for homogeneous meta-analyses and underlying OR=1.5 and moderate PB induced by p-value alongside meta-analysis sizes

**Figure A12** Measures of performance of the predicted effect for heterogeneous meta-analyses and underlying OR=1.5 and moderate PB induced by p-value alongside meta-analysis sizes

**Figure A8** Measures of performance of the predicted effect for meta-analyses simulated to have 30 studies, an underlying OR=1 & moderate PB induced by p-value alongside levels of heterogeneity

**Publication bias situation 4**

**Figure A13** Measures of performance of the predicted effect for meta-analyses simulated to have 30 studies, an underlying OR of 3 and severe PB induced by effect size alongside heterogeneity

**Figure A14** Measures of performance of the predicted effect for homogeneous meta-analyses and underlying OR=1 and severe PB induced by effect size alongside meta-analysis sizes

**Figure A15** Measures of performance of the predicted effect for heterogeneous meta-analyses and underlying OR=1 and severe PB induced by effect size alongside meta-analysis sizes

**Publication bias situation 5**

**Figure A16** Measures of performance of the predicted effect for meta-analyses simulated to have 30 studies, an underlying OR=1 & moderate PB induced by effect size alongside heterogeneity

**Figure A17** Measures of performance of the predicted effect for meta-analyses simulated to have 30 studies, an underlying OR of 3 and moderate PB induced by effect size alongside heterogeneity

**Figure A18** Measures of performance of the predicted effect for homogeneous meta-analyses and underlying OR=3 and moderate PB induced by effect size alongside meta-analysis sizes

**Figure A19** Measures of performance of the predicted effect for heterogeneous meta-analyses and underlying OR=3 and moderate PB induced by effect size alongside meta-analysis sizes
